# Dietary Guidelines Post Kidney Transplant: Is This the Missing Link in Recovery and Graft Survival?

**DOI:** 10.3389/ti.2025.14288

**Published:** 2025-04-03

**Authors:** Suzanne Schneider, Deborah Biggerstaff, Thomas M. Barber

**Affiliations:** ^1^ Directorate Applied Health, Warwick Medical School, University of Warwick, Coventry, United Kingdom; ^2^ Division of Biomedical Sciences, Warwick Medical School, University of Warwick, Coventry, United Kingdom; ^3^ Warwickshire Institute for the Study of Diabetes, Endocrinology and Metabolism, University Hospitals Coventry and Warwickshire, Coventry, United Kingdom

**Keywords:** kideny transplantation, nutrition guidelines, graft survival, post kidney transplant care, diet post transplant

## Abstract

The physiology of a transplanted kidney is affected from the moment it is separated from the donor. The risk of complications arising from surgery are highly associated with ischemic-reperfusion injury (IRI) due to the effects of hypoxia and oxidative stress during the procurement, preservation and reperfusion procedures. Hypoxia promotes the formation of reactive oxygen species (ROS) and it seems apparent that finding ways of optimising the metabolic milieu for the transplanted kidney would improve recovery and graft survival. Studies have demonstrated the benefits of nutrition and antioxidant compounds in mitigating the disturbance of energy supply to cells post-transplant and at improving long-term graft survival. Particularly in patients who may be nutritionally deficient following long-term dialysis. Despite the high incidence of allograft failure, a search of the literature and grey literature reveals no medical nutriti on therapy guidelines on beneficial nutrient intake to aid transplant recovery and survival. This narrative review aims to summarise current knowledge of specific macro and micronutrients and their effect on allograft recovery and survival in the perioperative period, up to 1-year post transplant, to optimise the metabolic environment and mitigate risk to graft injury.

## Introduction

The physiology of a transplanted organ is affected from the moment it is separated from the donor. The risk of complications arising from surgery are highly associated with ischemia-reperfusion injury (IRI) due to the effects of hypoxia and oxidative stress during the procurement, preservation and reperfusion procedures [1, 2]. As hypoxia promotes the formation of reactive oxygen species (ROS), it seems apparent that finding ways of improving antioxidant levels would optimise the milieu within which the transplanted organ is placed.

Nutrition is broadly accepted as playing a role in optimizing patients’ health pre- and post-transplant, and requirements for different nutrients change significantly as kidney function declines. Renal insufficiency is associated with significant changes in electrolyte handling and cellular balance of sodium, potassium, phosphate and calcium, all of which are biologically vital. Dietary restriction contributes significantly to reducing kidney disease progression in more advanced disease [3, 4]. Dietary restrictions limit the options of access to whole foods rich in these minerals, such as vegetables, dairy and nuts, which can cause patients to increase their intake of ultra processed foods (UPF). UPFs typically contain additives, preservatives, artificial sweeteners, and emulsifiers, with limited dietary fibre, all of which impact the biodiversity of the microbiome [5]. Kidney transplant recipients (KTR) are therefore at risk of nutritional deficiencies by the time they receive their donor organs, affecting antioxidant status, and potential imbalance in the gut microbiota, with increased production of uremic toxins [4, 6, 7].

### The Role of the Gut Microbiome

Evidence suggests that gut microbiota play an important role in the metabolism, storage, and expenditure of energy and nutrients, and play a pivotal role in host immunity, and metabolic function [8, 9]. The integrity of the gut microbiome therefore affects the host’s ability to absorb nutrients and regulate immunity [9].

Dysbiosis of intestinal flora is associated with complications in KTR, and many patients experience dysbiosis particularly in the first month post-transplant [10–12]. The causes of dysbiosis are multifactorial and can be assigned to the use of preparative regimens prior to transplantation as well as prophylactic antibiotics and immunosuppressant drugs [13]. Dysbiosis may influence graft outcomes, causing acute rejection, infection, renal fibrosis, and modification of drug metabolism [8, 14, 15].

Given the ability of the microbiota to influence isoimmunity and drug metabolism, data suggest that modifying the microbiota could contribute to more targeted immunosuppressive and post-transplant complication therapies, to improve graft survival and patients’ quality of life (QoL) [13, 16, 17]. Diet modification particularly the inclusion of prebiotic and prebiotic foods is beneficial in altering an abnormal microbiota to produce the host’s own antimicrobial substances, thereby improving immune function and graft survival [18, 19]. These prebiotic foods contain high amounts of fibre which serve as a food source for many of the gut microbiota, and a commensal partnership exists between the host and these bacteria [20].

While there is consensus on the increased risk of foodborne infection, especially in the first 6 months post-transplant, recommendations for the avoidance of consuming fresh fruit and vegetables vary across national guidelines [21, 22]. Several studies have questioned whether these protective diets provide any significant benefit in terms of infection rates, compared to a non-restrictive diet and may contribute to nutritional deficiencies [23, 24]. A common metric of gut health is the diversity of microbial species, and any acute changes can modify this composition within just 24 h [25, 26]. There is currently a lack of relative evidence referring to the microbiota in renal transplantation, with most studies conducted on animals [8, 27]. Research is therefore needed to understand the implications of chronic dysbiosis and its effect on graft survival in humans.

As nutrition is a vast subject, we acknowledge that this review does not cover all aspects of nutrition that might affect individual patients. We therefore focus specifically on nutrients that are highly monitored during ESKD to determine their effect on allograft health post-transplant and highlight the relevance of continued monitoring particularly in the critical early (up to 1 year) period post post-transplant.

## Materials and Methods

**FIGURE 1:**
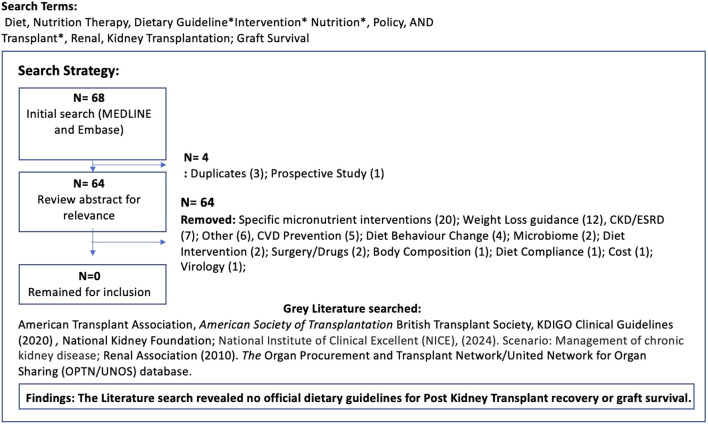
Literature review strategy: post kidney transplant dietary guidelines.

Published data were searched using the Medline National Library of Medicine, MEDLINE and Embase. No date restriction was applied, to broaden the search, however only English language papers were included. Search terms used included Diet, Nutrition Therapy, Dietary Guideline*Intervention* Nutrition*, Policy, AND Transplant*, Renal, Kidney Transplantation. 68 papers were identified, and after initial review of titles and abstracts for relevance, three duplicates were removed. A secondary review revealed no papers focused specifically on dietary guidelines post kidney transplant, although 20 covered individual macro and micronutrients which served as thematic insight for this paper. A grey literature search within the major national and international Kidney transplant organisations was also conducted to confirm whether any nutrition guidelines were available for post-transplant support. None were found (Figure 1). A narrative review, adopting a systematic synthesis of the available evidence of the individual macro and micronutrients was conducted with all papers reviewed by authors. Thematic analysis was identified by the primary author and confirmed by author 2 and 3. These themes will be discussed here.

## Results

Our findings show a paucity of specific dietary recommendations for KTR, and the studies currently available focus on single nutrient intakes, and not on the overall eating pattern. Considering that individuals do not typically consume nutrients in isolation it is challenging for single nutrient interventions to demonstrate conclusive effects and modifying dietary patterns as a whole may present a more realistic alternative or provide a complementary approach to single-nutrient interventions. We discuss these individual nutrients here, and demonstrate how the composition of the diet, particularly one that focuses on lower carbohydrate intake may be of increased relevance to graft survival.

### Macronutrients: Protein

Protein requirements change during various phases post-transplant. The first few weeks post-transplant are characterized by increased nutritional demands due to the associated stress of surgical insult to the body and the high doses of immunosuppressive medications [28]. During this critical phase large glucocorticoid doses cause accelerated protein catabolism to achieve positive Nitrogen balance and improve wound healing while conserving muscle [29, 30]. There are currently no agreed guidelines on a recommended protein intake for KTR, although a review by Chadban et al [29] recommends around 1.4 g/kg/day protein intake during the first 4 weeks post transplantation to reverse negative Nitrogen balance and increase muscle mass. This was also found protective in reducing the risk of increased fat mass and muscle loss up to 1 year post transplant. Once patients are on a maintenance diet, research suggests a distinction be made between diabetic and non-diabetic KTR patients, advocating slightly higher protein requirements in diabetic patients (0.8–0.9 g/kg/day vs. 0.6–0.8 g/kg protein/day) based on the beneficial effects of protein in stabilising blood glucose [31, 32].

KTR frequently suffer with severe fatigue, which ultimately affects quality of life (QoL), and the role of protein in muscle repair, energy metabolism and neurotransmitter production (such as dopamine and serotonin) are well documented [33, 34]. A cross-sectional study, involving 730 stable KTR [median age 58 years (IQR 48–65), 57% male] with a mean protein intake of 82.2 ± 21.3 g/d were assessed to examine the association of protein intake with fatigue and QoL. Moderate and severe fatigue were present in 254 (35%) and 245 (34%) of KTR. Higher protein intake was significantly associated with lower risk of moderate fatigue (OR 0.89 per 10 g/d; 95%CI 0.83–0.98, *p* = 0.01), severe fatigue (OR 0.85; 95%CI 0.78–0.92, *p* < 0.001) and was associated with higher physical component summary scores for QoL (β 0.74 per 10 g/d; 95%CI 0.39–1.09, *p* < 0.001) [35]. This suggests that higher protein intake is independently associated with lower risk of moderate and severe fatigue and better QoL in KTR. It is important to note that enhanced protein intake alone, without resistance training may limit this benefit, due to the anabolic stimulus that exercise provides in muscle maintenance [36].

Several studies have however found that restricting dietary protein in KTR with chronic allograft nephropathy or chronic rejection may be beneficial, with respect to kidney function; however, further research is needed to identify the magnitude of benefit and a safe level of intake for this patient group [37, 38].

### Carbohydrates

Metabolic disorders after kidney transplantation are common, and various dietary approaches have been studied regarding their effects on co-morbidity progression such as weight gain, hypertension, hyperlipidaemia, and insulin resistance [39]. Exposure to immunosuppressive medications such as glucocorticosteroids can cause or worsen preexisting hyperglycemia and weight gain [40–44], and regulating blood glucose has favourable downstream implications in slowing kidney disease progression [45–47].

As carbohydrates are the major contributor to post prandial hyperglycemia, increasing evidence highlights the benefits of very low-carbohydrate (ketogenic) diets to reduce inflammation, maintain euglycemia and weight, by improving satiety, reducing hyperglycemia and hyperinsulinemia. [48–50]. These diets are generally cautioned against for individuals with impaired kidney function, partly due to concerns about increased protein intake, which is associated with hyperfiltration and potentially, a decline in kidney function [51–53]. While classification of these diets differ greatly within the literature, differences are based on the proportion of total daily energy from carbohydrate and/or absolute carbohydrate intake [54]. Dietary analysis of very low carbohydrate studies usually report daily protein intake from 0.6 g/kg to 1.4 g/kg; which is below the high protein threshold (≥2.0 g/kg) believed to be of concern [55, 56].

The available literature on very low carbohydrate diets in KTR is scarce, although several studies recommend it as a therapy for preventing or assisting in recovery from ischemic and traumatic injuries [57–59]. The extensive topic of ketone body metabolism is beyond the scope of this article, but in brief, when following a keto diet (KD) or during fasting, fatty acids are relocated from adipocytes to liver cells, and transformed into the Acyl-CoA form, then transported to the liver to produce ketone bodies, which provide an alternative form of ATP energy [60]. Since disturbances in the energy supply of cells during ischemia cause a transient interruption of normal blood flow to the kidney, there is an increase in oxidative stress and inflammation [61]. Ketone bodies have demonstrated nephroprotective effects in IRI, due to their ability to suppress the concentration of proinflammatory factors, such as tumour necrosis factor alpha, interleukins including IL-6; IL-1β, IL-18, IFN, and decreased expression of the NF-κB and MCP-1 which induce the expression of various proinflammatory genes [57, 62]. The natriuretic and diuretic effect of the KD may also provide additional kidney protection by helping to alleviate sodium retention and improve systemic and glomerular blood pressure [63, 64].

As there is currently no agreement on isocaloric comparisons recommending a specific carbohydrate intake for KTR, clinicians are challenged to provide risk assessments and guidance [65]. While the KD implies an increased intake of fat, this definition is not standard across studies, and it is important to distinguish between the types of fat and their ratios in the overall diet, which will be discussed in the section below [66].

### Fats

There are currently no specific recommendations for dietary fat intake post kidney transplant, and patients are advised to follow the recommendations for the general population [31]. There is also no consensus on what the optimal ratio of n-6: n-3 polyunsaturated fats (PUFA) should be. Few studies investigate Essential Fatty Acid (EFA) deficiency in KTR, although low intakes have been attributed to renal hypertension, mitochondrial activity disorders, Cardiovascular Disease (CVD), type 2 diabetes, and decreased resistance to infection [67, 68].

Inflammation is part of the body’s immediate response to injury or infection, and it begins the immunological process of eliminating pathogens and toxins to repair damaged tissue [69]. Although inflammation is a normal response, when it occurs in an uncontrolled or inappropriate manner excessive damage and disease to the affected tissue(s) can ensue. Dyslipidaemia is a known risk factor for CVD and evidence suggests that KTR have significantly lower serum content of potentially beneficial Polyunsaturated Fatty Acids (PUFA) compared to CKD patients not on dialysis [70]. PUFAs help regulate the antioxidant signalling pathway and modulate inflammatory processes. Both Omega 6 and Omega 3 play a key part in balancing inflammation to achieve homeostasis. Several sources suggest that humans evolved on a diet that had a ratio of omega-6 to omega-3 EFA of about 1:1; whereas today, Western diets have a ratio of approximately 10:1 to 20:1 [71, 72]. While pro-inflammatory omega 6 plays an important part in host defence, by creating a hostile environment for microbes and later by initiating tissue repair, recovery, and maintenance of homeostasis, prolonged (unresolved) inflammation can cause tissue damage and metabolic changes [73]. By contrast, Omega −3 (n-3) have shown improved renal and cardiovascular prognosis, and protective benefits against inflammation and overall mortality in KTR, due to their antithrombotic, anti-inflammatory, and antiarrhythmic effects [74–77].

In one study investigating the effects of n-3 PUFA supplementation on kidney allograft function and lipid profile, 60 long-term, first time KTR were assigned to 2 groups: a CON group (*n* = 28), who continued with their usual diet, and the DIET group (*n* = 32), who followed an n-3-PUFA rich diet for 6 months to investigate changes in n-3 PUFAs intake; the n-6: n-3 PUFAs ratio, systemic inflammation markers, and renal function. At 3 and 6 months the DIET group had significantly higher n-3 PUFA levels and a markedly lower n-6: n-3 PUFA ratio than baseline. This group also had reduced systemic inflammation with decreased plasma total cholesterol, triglycerides, C-reactive protein, and decreased interleukin (IL)-6. While eGFR remained unchanged, this group also experienced 50% reduction in proteinuria and microalbuminuria compared to baseline [78].

Further clinical studies are needed to confirm beneficial ratios of n6: n3, particularly in the initial weeks and months post-transplant, to gauge the positive effects of controlled inflammation as part of the healing process, and the protective effects of n3 in renal function long term.

### Micronutrients

#### Sodium

The literature regarding sodium intake and hypertension in KTRs is scarce and gaps in knowledge still exist on the exact amount needed to optimize graft outcomes and reduce the risk of CVD. This is mostly due to the lack of clarification on the best methods to measure sodium intake; and the often-complex co-morbidities experienced by KTR. The 2012 KDIGO Clinical Practice Guideline recommend a salt intake to <90 mmol (<2 g)/day of sodium (corresponding to 5 g of sodium chloride) for CKD patients with high blood pressure, the same as for the general population [79]. The supporting evidence for this recommendation is of low quality as it references only an adequate intake for adults aged 19–50 years, “based on meeting sodium needs of apparently healthy individuals.” This infers that the guidelines are relevant to those who are moderately active, live in a temperate climate and have no metabolic diseases or compromised kidney function, which does not apply to KTR.

A 2024 literature review by Afsar et al investigating sodium intake and renal transplantation showed continued inconsistencies [80]. Some studies found no relationship between sodium intake and hypertension [81–83] while others found a positive association, although these studies were conducted on rats [84, 85]. Contrasting views also found no association between sodium intake and proteinuria/albuminuria in graft function [86] while others showed a positive association [87, 88].

Numerous studies highlight the effect of insulin on renal sodium transport and metabolism; and demonstrate that individuals with arterial hypertension have reduced insulin sensitivity and hyperinsulinemia, compared to subjects with normal blood pressure [89–91].

As a mineralocorticoid, insulin plays an important role in sodium balance, particularly in conditions of elevated circulating plasma insulin concentrations. Plasma insulin stimulates sodium reabsorption by the distal nephron segments, causing hyperfiltration and a rise in intra-glomerular pressure [64]. As carbohydrates are the major contributor to post prandial hyperglycemia and subsequent insulin secretion, it seems logical that to achieve sodium balance and insulin homeostasis it is necessary to modify the diet, by substitution of carbohydrates with lower carb alternatives [45, 92].

Prospective long-term, randomised controlled studies of the effect of the KD in KTR are warrened specifically investigating their effect on electrolyte imbalance, hyperfiltration and the downstream effects on allograft function [93].

#### Potassium

Disturbances of potassium balance is a frequent complication among KTR notably immediately post-transplant, and in those with suboptimal graft function and higher calcineurin inhibitor levels [94, 95]. Despite the high incidence and potential life-threatening implications, consensus on potassium management in KTR is lacking – with post-transplant medications and dietary induced hyperkalemia associated with decreased glomerular filtration rates and impaired sodium delivery in the distal nephron [96].There is currently a lack of research on the specific consequences of untreated hyperkalemia to KTR, although insights from CKD populations highlight the importance of maintaining normal serum K+ concentrations particularly in IRI post transplantation, where cells experience metabolic shifts that lead to the inhibition of sodium-potassium ATPase. This inhibition disrupts ion homeostasis, contributing to increased ROS production and subsequent cellular damage [97]. Potassium also helps regulate the inflammatory response by influencing the activation of immune cells and the release of cytokines. Post transplant K+ balance is also vital for cardiovascular and renal outcomes [98–100].

Dietary guidelines for potassium vary greatly across the literature and none are specifically directed at KTR (Table 1). KTR that do experience hyperkalemia are frequently advised to avoid high-potassium plant-based foods, although the associated effectiveness is weak as the bioavailability and metabolism of K+ is naturally influenced by the other nutrients consumed [105–107]. K+ from plant-based sources in particular have proved beneficial, as they provide alkali and antioxidant vitamins, trace elements and fibre, which promotes intracellular entry and excretion of K+ in stool by increasing faecal volume [108]. As constipation is a frequent symptom in KTR, restricting fibre-rich foods can impact intestinal microbiota composition and increase the risk of metabolic acidosis and inflammation [109–111].

**TABLE 1:** Variations in potassium recommendations across the general and CKD population.

US food and nutrition board (IOM. 2005) [101]	The World Health Organization (WHO, 2012) [102]	K/DOQI, National Kidney Foundation (2000) [103]	Comprehensive review by Kalantar-Zadeh et al., (2017) [104]
4.7 g (120 mmol) per day in healthy adults	3.9 g (100 mmol) per day or at least 90 mmol/day (3,510 mg/day) in healthy adults. No specific guidelines for kidney disease (CKD stage 1–5)	Unrestricted potassium intake in non-dialysis dependent patients with CKD stage 1–5 In hemodialysis patients, up to 2.7–3.1 g/day and in peritoneal dialysis patients up to 3–4 g/day	Intake of 4.7 g/day in the early stages of CKD without risk of hyperkalaemia, but a dietary potassium restriction of less than 3 g (77 mmol) per day in CKD patients prone to hyperkalaemia

### The Influence of Insulin on K+ Balance

Multiple compensatory mechanisms are enhanced in CKD to maintain potassium homeostasis. Insulin facilitates the uptake of K+ into the cells by activating the Na+/Ka+-ATPas pump [112].

In hyperglycemia, elevated glucose leads to osmotic diuresis, causing significant loss of water and electrolytes, including K+, resulting in an apparent elevation of serum K+ while depleting cellular stores [100]. Studies show that reducing insulin requirements through reduced carbohydrate consumption improves insulin sensitivity which in turn helps to stabilise K+ levels [100, 113].

As new onset diabetes after transplant (NODAT) is a common complication occurring in up to 50% of KTR, there is a need for more specific dietary guidelines to optimise insulin balance [114]. Latest guidelines from *KDIGO (2023*) [115] contain no references to dietary recommendations for K+, despite commendation that “a healthy diet should be maintained.”

#### Vitamin D

Numerous studies demonstrate a high prevalence of vitamin D deficiency in KTR, likely due to the effects of immunosuppressive regimens and renal function impairment which affects the ability of the kidneys to convert 25-hydroxyvitamin D [25(OH)D] into 1,25-dihydroxyvitamin D [1,25(OH)_2_D] (the active form), and advice that transplant recipients avoid sunlight to minimise the risk of skin cancer [116, 117]. Existing research in KTR highlights the challenges of achieving adequate vitamin D through diet alone and that even after successful kidney transplantation, the activity of 25-dihydroxyvitamin D may not fully normalize [118]. Supplementation is therefore considered more effective, particularly in vitamin D deficiency and excessive immune inflammation [119–121].

Vitamin D has an established function in immunological health, due to its role in calcium homeostasis and restoring mitochondrial membranes by regulating intracellular Ca^2+^ concentrations to decease ROS production in IRI [122]. Several studies show that low levels of 25(OH) vitamin D can have deleterious effects on renal allograft health and increase the risk of NODAT [123–127]. Severe vitamin D deficiency is defined as having a serum 25OHD concentration of <10 ng/mL (25 nmol/L) [128]). KDIGO (2020) guidelines for patients with CKD (stage 1–5) suggest that vitamin D deficiency and insufficiency be treated using strategies recommended for the general population. However, a 2015 study of 289 KTR showed that vitamin D status is negatively affected by calcineurin inhibitors (specifically tacrolimus), the most commonly used immunosuppressant, but not experienced in newer mTOR inhibitors, such as sirolimus and rapamycin [117]. Appropriate management of immunosuppression therapy and monitoring of vitamin D status in KTR therefore warrants a more careful and individualised approach compared to the general population.

It is worth noting that studies on vitamin D deficiency in KTR only proves association and adverse outcomes, but not causality. Continuation of the primary disease (i.e.,: presence of CKD) or a *de novo* disease in the kidney graft could also contribute to proteinuria, worse kidney function and mortality [120]. Larger prospective and interventional RCTs are needed to fully assess the influence of vitamin D on post-transplant outcomes, and the benefits of long-term supplementation.

#### Vitamin C

KTR are prone to vitamin C deficiency mostly due to the potential remnant long-term effects of dialysis and higher requirements due to the enhanced pro-oxidative and pro-inflammatory status following IRI [129, 130]. Vitamin C is a powerful biological antioxidant which serves as cofactor for several enzymes involved in anti-inflammatory responses, collagen hydroxylation, carnitine and catecholamine biosynthesis [131]. KTR with low levels of vitamin C are therefore at increased risk of poor wound healing and infection immediately post-surgery, and face higher risk of long-term graft failure, due to reduced biosynthesis of collagen and regulatory T cells [132–134].

In a trial assessing Vitamin C in 598 KTR at 3-, 6-, 12-, 24-, and 60-month post-transplantation, Vitamin C deficiency was defined as plasma vitamin C ≤ 28 μmol/L [135]. At all measurement points, KTR had lower plasma vitamin C than potential donors (30–41 μmol/L vs. 58 μmol/L), with deficiency ranging from 46% (6-month post-transplantation) to 30% (≥1-year post-transplantation). Dietary vitamin C intake and vitamin C supplementation were associated with lower odds (OR per 100 mg/day 0.38, 95% CI 0.24–0.61 and OR 0.21, 95% CI 0.09–0.44, respectively). This suggests a strong need for vitamin C analysis and potential supplementation, particularly in individuals with delayed graft function.

Supplemental doses of vitamin C of 90 mg to 3 g/day are considered safe, with mild adverse effects, including gastrointestinal disturbances [136]. Studies on long term, high dose supplementation show increased risk of kidney stones (particularly in males with renal insufficiency), due to increased urinary excretion of oxalate [137, 138]. This risk is not replicated in dietary vitamin C due to the saturable absorption of vitamin C from the gastrointestinal tract [139], and the fact that most dietary sources (such as fruit and vegetables) include a high water content.

### The Effect of Vitamin C on Delayed Graft Function

In IRI, endothelial cells are activated by the upregulation of pro-inflammatory cytokines. Vitamin C reduces inflammation and endothelial permeability by increasing pro-inflammatory cytokines and phagocytes that contribute to ROS reduction [41, 42, 140, 141]. In a small (19 participant) double-blinded RCT, investigating the effect of vitamin C on delayed graft function (DGF), KTR in the treatment group received an intravenous vitamin C infusion (70 mg/kg diluted in 0.45% saline), with the control group receiving only the dilute solution. The incidence of DGF was not significantly different between the groups after a single dose of vitamin C, although the duration of DGF was substantially shorter in the vitamin C group than the placebo group (7.33 ± 5.68 versus 19.66 ± 0.57 days; P = 0.02) [142]. It is important to note that this study did not include the nutrition status of participants and therefore those with higher deficiency rates may have experienced more dramatic outcomes. Additionally, considering the short half-life of the vitamin and the nature of surgical delays, a bolus intravenous dose of vitamin C may have produced more accurate results.

While vitamin C supplements, particularly in the first month post-transplant might provide a safer and more measurable form of intake, the sodium content of vitamin C preparations should be considered, particularly in sodium-restricted patients [70, 136].

## Discussion

Our research showed a positive association between poor nutrition status and impact on allograft recovery and survival. There is consensus grounded in evidence that transplant patients have distinct nutritional needs, with many KTR being nutritionally deficient by the time they receive their donor organ, placing them at increased risk of IRI, graft failure and mortality [143, 144]. There are currently limited studies investigating the longitudinal dietary intake of KTR, yet as this group are still considered a subset of patients with CKD they remain at high risk for progression to dialysis and mortality [145]. Our research highlights the difficulty of investigating the effect of individual macro- and micronutrients on allograft health although there is sufficient evident to highlight the negative impact of higher glycaemic diets, due to the downstream effects on renal sodium transport and the effects of hyperinsulinemia on intra-glomerular pressure. While most metabolic disorders post-transplant cannot be modified, diet and obesity are two factors that can safely be manipulated particularly in preventing metabolic disorders such as NODAT and CVD [146]. Obesity is associated with the prevalence and progression of CKD and low carbohydrate diets are recognised as an effective treatment in weight loss [147, 148]. In a context where the prevalence of nutrition-related health conditions is growing, there is an urgent need for nutrition education for physicians, who receive on average less than 24 contact hours of nutrition instruction across the medical degree [149]. Many do not feel comfortable, confident, or adequately prepared to provide nutrition counselling and this gap in knowledge is contributing to poorer patient outcomes [150, 151].

Findings on PUFA intake demonstrated that the beneficial effects of anti-inflammatory n-3 depend primality on the dietary n6: n3 ratio. There is no consensus in the literature on what constitutes an optimal ratio, but the benefits of PUFA homeostasis demonstrate that for KTR there is a need for further research particularly to understand whether a higher n-6 ratio in the period immediately post-transplant might enhance immunity, tissue repair and recovery. Longer term maintenance strategies which include a reduction in carbohydrates will naturally have a higher percentage of protein and fat to compensate for the reduced calories [54] and future guidelines are needed to guide patients on optimal composition of dietary fats at various stages post-transplant.

Evidence on protein requirements post-transplant remain contentious and updated research is needed to ascertain a safe level of intake. It is likely that future guidance on protein intake will be based on prevailing renal function and the magnitude of benefit of higher intake to counteract protein catabolism and muscle protein wastage. Literature on hypovitaminosis D in KTR suggest that low sunlight exposure and the accelerated catabolism of vitamin D secondary to glucocorticoid use increases the risk of renal allograft failure and development of NODAT [123]. Evidence suggests that the general population in the UK are deficient of vitamin D, specifically in the winter months [122]. There is therefore a case for individualised monitoring and replacement therapy in this group.

*KDIGO clinical guidelines (KDIGO, 2020*) recognise that immunosuppression and graft function are only one component of healthcare, yet it makes no mention of the role of nutrition on allograft health and survival. This research demonstrates that monitoring of nutritional status post-transplant should be a clinical priority, with personalised dietary recommendations and provision for self-management strategies.

## Conclusion

Despite significant medical advances over the last few decades, kidney transplants frequently do not function for the lifetime of the recipient, with more than a third of kidney grafts failing within 10 years following transplantation [152, 153]. It is widely known that nutrition influences all metabolic disease, health and recovery and more specific research is needed on the beneficial effect of targeted nutrition in establishing an optima metabolic milieu for the transplanted organ to thrive. Clear guidelines which are accessible to patients and clinicians are we suggest, essential since these will provide the missing link in post-transplant care.

## References

[1] Mauerhofer C Grumet L Schemmer P Leber B Stiegler P. Combating Ischemia-Reperfusion Injury With Micronutrients and Natural Compounds During Solid Organ Transplantation. Data of Clinical Trials and Lessons of Preclinical Findings. Intl J Mol Sci (2021) 22:10675. 10.3390/ijms221910675 PMC8508760 34639016

[2] Alvarez J del Barrio R Arias J Ruiz F Iglesias J de Elías R Non-heart-beatine Donors From the Streets: An Increasing Donor Pool Source. Transplantation (2000) 70(02):314–7. 10.1097/00007890-200007270-00014 10933156

[3] Mazzaferro S Martini N Cannata-Andia J Cozzolino M Messa P Rotondi S Focus on the Possible Role of Dietary Sodium, Potassium, Phosphate, Magnesium and Calcium on CKD Progression. J Clin Med (2021) 10:958. 10.3390/jcm10050958 33804573 PMC7957473

[4] Clase CM Carrero JJ Ellison DH Grams ME Hemmelgarn BR Jardine MJ Potassium Homeostasis and Management of Dyskalemia in Kidney Diseases: Conclusions From a Kidney Disease: Improving Global Outcomes (KDIGO) Controversies Conference. Kidney Int Ki (2020) 97(1):42–61. 10.1016/j.kint.2019.09.018 31706619

[5] Whelan K Bancil AS Lindsay JO Chassaing B. Ultra-processed Foods and Food Additives in Gut Health and Disease. Nat Rev Gastroent Hepatol (2024) 21:406–27. 10.1038/s41575-024-00893-5 38388570

[6] Cupisti A Avesani CM D’Alessandro C Garibotto G. Nutritional Management of Kidney Diseases: An Unmet Need in Patient Care. J Nephrol (2020) 33:895–7. 10.1007/s40620-020-00829-7 32785842

[7] Anupama SH Abraham G Parthasarathy R Anupama P Mathew M Malnutrition in Kidney Transplantation: Our Experience and Review of the Literature. Indian Journal of Transplantation (2020). 10.4103/ijot.ijot_40_19

[8] Rowland I Gibson G Heinken A Scott K Swann J Thiele I Gut Microbiota Functions: Metabolism of Nutrients and Other Food Components. Eur J Nutr (2017) 57(1):1–24. 10.1007/s00394-017-1445-8 28393285 PMC5847071

[9] Wu HJ Wu E. The Role of Gut Microbiota in Immune Homeostasis and Autoimmunity. Gut Microbes (2012;2012) 3(1):4–14. 10.4161/gmic.19320 PMC3337124 22356853

[10] Chen Z Chang X Ye Q Gao Y Deng R. Kidney Transplantation and Gut Microbiota. Clin Kidney J (2024) 17:8. 10.1093/ckj/sfae214 PMC11336673 39170931

[11] Chao KT Lin YK Chen LK Huang P Yi-Chung H Role Fot He Gut Microbiota and Their Metabolites in Hemodialysis Patient. Int J Med Sci (2023) 20(6):725–36. 10.7150/ijms.82667 37213669 PMC10198149

[12] Guirong Ye Minjie Z Lixin YU Junsheng YE Lin Y Lisha S. Gut Microbiota in Renal Transplant Recipients, Patients With Chronic Kidney Disease and Healthy Subjects. Nan Fang Yi Ke Da Xue Xue Bao (2018) 38:1401–8. 10.12122/j.issn.1673-4254.2018.12.01 30613005 PMC6744200

[13] Chong F Koh A. The Gut Microbiota in Transplant Patients. Blood Rev (2020) 39:100614. 10.1016/j.blre.2019.100614 31492463 PMC7002184

[14] Salvatori M Tsalouchos A. The Microbiota and Kidney Transplantation: Influence on the Graft. Eur Med J Urol (2021) 9(1):95–106.

[15] Yu DHYN Lian ZH Fa YQ. The Alteration Human of Gut Microbiota and Metabolites before and after Renal Transplantation. Microb Pathog (2021) 160:105191. 10.1016/j.micpath.2021.105191 34571151

[16] Wang J Li X Wu X Wang Z Zhang C Cao G Gut Microbiota Alterations Associated With Antibody-Mediated Rejection after Kidney Transplantation. Appl Microbiol Biotechnol (2021) 105:2473–84. 10.1007/s00253-020-11069-x 33625548

[17] Guo Y Lee H Edusei E Albakry S Jeong H Lee JR. Blood Profiles of Gut Bacterial Tacrolimus Metabolite in Kidney Transplant Recipients. Transpl Direct (2020) 6:601. 10.1097/TXD.0000000000001052 PMC7591115 33134481

[18] Melini F Melini V Luziatelli F Ficca AG Ruzzi M. Health-Promoting Components in Fermented Foods: An Up-To-Date Systematic Review. Nutrients (2019) 11:1189. 10.3390/nu11051189 31137859 PMC6567126

[19] Tarvainen M Fabritius M Yang B. Determination of Vitamin K Composition of Fermented Food. Food Chem (2019) 275:515–22. 10.1016/j.foodchem.2018.09.136 30724228

[20] Barber TM Kabisch S Pfeiffer AF Weickert MO. The Health Benefits of Dietary Fibre. Nutrients (2020) 12(10):3209. 10.3390/nu12103209 33096647 PMC7589116

[21] American Society of Transplantation. Guidelines for Post-kidney Transplant Management in the Community Setting. Am Soc Transplant (2009).

[22] National Health Service, UK (NHS). In: Neo D, editor. Eating Well after Your Transplant. (2023). (NHS, University Hospitals Coventry and Oxfordshire). HIC/LFT/503/07. 1-52024.

[23] Pullen LC. Protective Diet Does Not Benefit Patients Receiving HT Compared to Non-restricted Diet. Am Soc Hematol (2023).

[24] Todd J Schmidt M Christian J Williams R. The Low-Bacteria Diet for Immunocompromised Patients. Reasonable Prudence or Clinical Superstition? Cancer Pract (1999) 7:205–7. 10.1046/j.1523-5394.1999.74009.x 10687586

[25] Butler MJ Perrini AA Eckel LA. The Role of the Gut Microbiome, Immunity, and Neuroinflammation in the Pathophysiology of Eating Disorders. Nutrients (2021) 13(2):500. 10.3390/nu13020500 33546416 PMC7913528

[26] Kleiman SC Watson HJ Bulik-Sullivan EC Huh EY Tarantino LM Bulik CM The Intestinal Microbiota in Acute Anorexia Nervosa and During Renourishment: Relationship to Depression, Anxiety, and Eating Disorder Psychopathology. Psychosomatic Med (2015) 7(9):969–81. 10.1097/PSY.0000000000000247 PMC4643361 26428446

[27] Kalantar-Zadeh K Tortorici A Joline LT Chen JLT Kamgar M Lau WL Moradi H Dietary Restrictions in Dialysis Patients: Is There Anything Left to Eat? Seminary Dial (2015) 28(2):159–68. 10.1111/sdi.12348 PMC4385746 25649719

[28] Shah N Sirsat R Trivedi M Kothari J Almeida A The Effect of Higher and Lower Protein Intakes on Nitrogen Balance in Rrnal Transplant Recipients With Underlying Graft Dysfunction. Saudi J Kidney Dis Transplant (2022) 33(2):236–44. 10.4103/1319-2442.379021 37417175

[29] Chadban S Chan M Fry K Patwardhan A Ryan C Trevillian P The CARI Guidelines. Protein Requirement in Adult Kidney Transplant Recipients. Nephrol Carlton (2010) 15(1):S68–S71. 10.1111/j.1440-1797.2010.01238.x 20591048

[30] Hoy WE Sargent JA Hall D McKenna BA Pabico RC Freeman RB Protein Catabolism During the Postoperative Course After Renal Transplantation. Am J Kidney Dis (1985) 5:186–90. 10.1016/s0272-6386(85)80049-4 3883760

[31] Teger NB. Owner's Manual: Nutrition Care for Your Kidney Transplant. J Ren Nutr (2019) 29:249–55. 10.1053/j.jrn.2018.02.006 29807683

[32] Campbell A Rains TM. Dietary Protein Is Important in the Practical Management of Diabetes. Diabetes Educator (2015) 41(4):486–93. 10.3945/jn.114.194878 25527675

[33] Fernstrom JD. Large Neutral Amino Acids: Dietary Effects on Brain Neurochemistry and Function. Amino Acids (2013) 45(3):419–30. 10.1007/s00726-012-1330-y 22677921

[34] Wolfe RR. The Underappreciated Role of Muscle in Health and Disease. The Am J Clin Nutr (2006) 84(3):475–82. 10.1093/ajcn/84.3.475 16960159

[35] Neto AWG Boslooper-Meulenbelt K Geelink M van Vliet IMY Post A Joustra ML Protein Intake, Fatigue and Quality of Life in Stable Outpatient Kidney Transplant Recipients. Nutrients (2020) 2020(12):2451. 10.3390/nu12082451 PMC7469059 32824065

[36] Hendriks F Smeets JS van der Sande F Kooman JP van Loon LJC. Dietary Protein and Physical Activity Interventions to Support Muscle Maintenance in End Stage Renal Disease Patients on Hemodialysis. Nutrients (2019) 11(12):2972. 10.3390/nu11122972 31817402 PMC6950262

[37] Chan M Patwardhan A Ryan C Trevillian P Chadban S Westgarth F Evidence Based Guidelines for the Nutiritonal Management of Adult Kidney Transplant Recipients. J Ren Nutr. (2011) 21:47–5147. 10.1053/j.jrn.2010.10.021 21195919

[38] Salahudeen AK Hostetter TH Raatz SK Rosenberg ME. Effect of Dietary Protein in Patients with Chronic Renal Transplant Rejection. Kidney Int (1992) 183–90. 10.1038/ki.1992.25 1593854

[39] Radic J Vuckovic M Gelemanovic A Kolak E Bučan Nenadić D Begović M Associations Between Advanced Glycation End Products, Body Composition and Mediterranean Diet Adherence in Kidney Transplant Recipients. Int J Environ Res Public Health (2022) 19(17):11060. 10.3390/ijerph191711060 36078776 PMC9518364

[40] Gorska M Kurnatowska I. Nutrition Disturbances and Metabolic Complications in Kidney Transplant Recipients: Etiology, Methods of Assessment and Prevention-A Review. Nutrients (2022) 14(23):4996. 10.3390/nu14234996 36501026 PMC9738485

[41] Sotler R Polisak B Dahmane R Jukić T Pavan Jukić D Rotim C Prooxidant Activities of Antioxidants and Their Impact on Health. Acta Clin Croat (2019) 58(4):726–36. 10.20471/acc.2019.58.04.20 32595258 PMC7314298

[42] Dennis JM Witting PK. Protective Role for Antioxidants in Acute Kidney Disease. Nutrients (2017) 9:718. 10.3390/nu9070718 28686196 PMC5537833

[43] Dunford EC Riddell MC. The Metabolic Implications of Glucocorticoids in a High-Fat Diet Setting and the Counter-effects of Exercise. Metabolites (2016) 6:44. 10.3390/metabo6040044 27929385 PMC5192450

[44] Geer EB Islam J Buettner C. Mechanisms of Glucocorticoid-Induced Insulin Resistance: Focus on Adipose Tissue Function and Lipid Metabolism. Endocrinol Metab Clin N Am (2014) 43:75–102. 10.1016/j.ecl.2013.10.005 PMC3942672 24582093

[45] Athinarayanan SJ Roberts CGP Uangala C Shetty GK McKenzie AL Weimbs T The Case for a Ketogenic Diet in the Management of Kidney Disease. BMJ Open Diab Res Care (2024) 12:e004101. 10.1136/bmjdrc-2024-004101 PMC11057262 38677719

[46] Leow Z Guelfi K Davis E Jones T Fournier P. The Glycaemic Benefits of a Very‐Low‐Carbohydrate Ketogenic Diet in Adults With Type 1 Diabetes Mellitus May Be Opposed by Increased Hypoglycaemia Risk and Dyslipidaemia. Diabetic Med (2018) 35(9):1258–63. 10.1111/dme.13663 29737587

[47] Krebs JD Strong AP Cresswell P Reynolds AN Hanna A Haeusler S. A Randomised Trial of the Feasibility of a Low Carbohydrate Diet vs Standard Carbohydrate Counting in Adults With Type 1 Diabetes Taking Body Weight into Account. Asia Pac J Clin Nutr (2016) 25(1):78–84. 10.6133/apjcn.2016.25.1.11 26965765

[48] Sumithran P Prendergast LA Delbridge E Purcell K Shulkes A Kriketos A Ketosis and Appetite-Mediating Nutrients and Hormones After Weight Loss. Eur J Clin Nutr (2013) 67(7):759–64. 10.1038/ejcn.2013.90 23632752

[49] Veldhorst M Smeets A Soenen S Hochstenbach-Waelen A Hursel R Diepvens K Protein-Induced Satiety: Effects and Mechanisms of Different Proteins. Physiol and Behav (2008) 94(2):300–7. 10.1016/j.physbeh.2008.01.003 18282589

[50] Heinemann L. Variability of Insulin Absorption and Insulin Action. Diabetes Technol and Ther (2002) 4(5):673–82. 10.1089/152091502320798312 12450450

[51] ElSayed NA Aleppo G Aroda VR Bannuru RR Brown FM Bruemmer D 5. Facilitating Positive Health Behaviors and Well-Being to Improve Health Outcomes: Standards of Care in Diabetes-2023. Diabetes Care (2023) 46:S68–96. 10.2337/dc23-S005 36507648 PMC9810478

[52] Jhee H Kee YK Park S Kim H Park JT Han SH High-protein Diet With Renal Hyperfiltration Is Associated With Rapid Decline Rate of Renal Function: A Community-Based Prospective Cohort Study. Nephrol Dial Transpl (2020) 35:98–106. 10.1093/ndt/gfz115 31172186

[53] Esmeijer K Geleijnse JM de Fijter JW Kromhout D Hoogeveen EK. Dietary Protein Intake and Kidney Function Decline after Myocardial Infarction: The Alpha Omega Cohort. Nephrol Dial Transpl (2020) 35:106–15. 10.1093/ndt/gfz015 PMC8205500 30768201

[54] Schneider S Biggerstaff D Barber TM. Helpful or Harmful? The Impact of the Ketogenic Diet on Eating Disorder Outcomes in Type 1 Diabetes Mellitus. Expert Rev Endocrinol Metab (2022) 17:319–31. 10.1080/17446651.2022.2089112 35748612

[55] Westman EC Yancy WS Mavropoulos JC Marquart M McDuffie JR. The Effect of a Low-Carbohydrate, Ketogenic Diet Versus a Low-Glycemic Index Diet on Glycemic Control in Type 2 Diabetes Mellitus. Nutr Metab Lond (2008) 5:36. 10.1186/1743-7075-5-36 19099589 PMC2633336

[56] Yancy WS Olsen MK Guyton JR Bakst RP Westman EC. A Low-Carbohydrate Ketogenic Diet Versus a Low-Fat Diet to Treat Obesity and Hyperlipidaemia. Ann Intern Med (2004) 140:769–77. 10.7326/0003-4819-140-10-200405180-00006 15148063

[57] Rojas-Morales PDLPC Ramos A Sánchez-Tapia M Silva-Palacios A Cano-Martínez A González-Reyes S A Ketogenic Diet Attenuates Acute and Chronic Ischemic Kidney Injury and Reduces Markers of Oxidative Stress and Inflammation. Life Sci (2022) 289:120227. 10.1016/j.lfs.2021.120227 34921866

[58] Guo M Wang X Zhao Y Yang Q Ding H Dong Q Ketogenic Diet Improves Brain Ischemic Tolerance and Inhibits NLRP3 Inflammasome Activation by Preventing Drp1-Mediated Mitochondrial Fission and Endoplasmic Reticulum Stress. Front Mol Neurosci (2018) 11:86. 10.3389/fnmol.2018.00086 29662437 PMC5890101

[59] Xu K Ye L Sharma K Jin Y Harrison MM Caldwell T Diet-induced Ketosis Protects Against Focal Cerebral Ischemia in Mouse. Adv Exp Med Biol (2017) 977:205–13. 10.1007/978-3-319-55231-6_28 28685447

[60] Makievskaya CI Pookov V Andriannova N. Ketogenic Diet and Ketone Bodies against Ischemic Injury: Targets, Mechanisms, and Therapeutic Potential. Intl J Mol Sci (2023) 24(3):2576. 10.3390/ijms24032576 PMC9916612 36768899

[61] Eltzschig HK Eckle T. Ischemia and Reperfusion—From Mechanism to Translation. Nat Med (2011) 17:1391–401. 10.1038/nm.2507 22064429 PMC3886192

[62] Tajima T Yoshifuji A Matsui A Itoh T Uchiyama K Kanda T β-Hydroxybutyrate Attenuates Renal Ischemia-Reperfusion Injury Through its Anti-Pyroptotic Effects. Kidney Int (2018) 95:1120–37. 10.1016/j.kint.2018.11.034 30826015

[63] Bovée DMCC Zietse R Danser AHJ Mirabito Colafella KM Hoorn EJ. Salt-sensitive Hypertension in Chronic Kidney Disease: Distal Tubular Mechanisms. Am J Physiol Ren Physiol (2020) 319:F729–45. 10.1152/ajprenal.00407.2020 32985236

[64] Unwin DJ Murray SW Delon C Brady AJ. Substantial and Sustained Improvements in Blood Pressure, Weight and Lipid Profiles from a Carbohydrate Restricted Diet. Int J Environ Rest Public Health (2019) 2019:16. 10.3390/ijerph16152680 PMC6695889 31357547

[65] Gray A Threlkeld RJ. Nutritional Recommendations for Individuals With Diabetes. (2015).

[66] De Roos Nm Bots ML Katan MB. Replacement of Dietary Saturated Fatty Acids by Trans Fatty Acids Lowers Serum HDL Cholesterol and Impairs Endothelial Function in Healthy Men and Women. Arterioscler Thromb Vasc Biol (2001) 21:1233–7. 10.1161/hq0701.092161 11451757

[67] Shetty SSKS Kumari S. Fatty Acids and Their Role in Type‑2 Diabetes (Review). Exp Ther Med (2021) 22(1):706. 10.3892/etm.2021.10138 34007315 PMC8120551

[68] Simopoulos AP. Simopoulos AP Essential Fatty Acids in Health and Chronic Disease. Am J Clin Nutr (1999) 70:560s–569s. 10.1093/ajcn/70.3.560s 10479232

[69] Calder PC. Polyunsaturated Fatty Acids and Inflammation. Prostaglandins Leukot Essent Fatty Acid (2006) 75:197–202. 10.1016/j.plefa.2006.05.012 16828270

[70] Sikorska-Wisniewska M Sledzinski T Mika A Czaplinska M Malgorzewicz S Debska-Slizien A Disorders of Serum Polyunsaturated Fatty Acids in Renal Transplant Patients. Transplant Proc (2020) 1–7. 10.1016/j.transproceed.2020.01.106 32334793

[71] Simopoulos M. The Omega 6/ômega-3 Fatty Acid Ratio: Health Implications. (2001) VOL 17 N 5 Sept-Oct 2010 267.

[72] Simopoulos A. Evolutionary Aspects of Diet: The Omega-6/Omega-3 Ratio and the Brain. Mol Neurobiol (2011) 44(2):203–15. 10.1007/s12035-010-8162-0 21279554

[73] Djuricic I Calder PC. Beneficial Outcomes of Omega-6 and Omega-3 Polyunsaturated Fatty Acids on Human Health: An Update for 2021. Nutrients (2021) 13(7):2421. 10.3390/nu13072421 34371930 PMC8308533

[74] Rund KM Peng S Greite R Claaßen C Nolte F Oger C Dietary Omega-3 PUFA Improved Tubular Function after Ischemia Induced Acute Kidney Injury in Mice but Did Not Attenuate Impairment of Renal Function. Prostaglandins Other Lipid Mediat (2020) 146:106386. 10.1016/j.prostaglandins.2019.106386 31698142

[75] Calder PC. Functional Roles of Fatty Acids and Their Effects on Human Health. J Parent Enter Nutr (2015) 39(Suppl. 1):S18e32. 10.1177/0148607115595980 26177664

[76] Eide IA Reinholt FP Jenssen T Hartmann A Schmidt EB Åsberg A Effects of Marine N-3 Fatty Acid Supplementation in Renal Transplantation: A Randomized Controlled Trial. Am J Transpl Off J Am Soc Transpl Am Soc Transpl Surg (2019) 19:790–800. 10.1111/ajt.15080 30125457

[77] Mozaffarian DWJH. Omega-3 Fatty Acids and Cardiovascular Disease: Effects on Risk Factors, Molecular Pathways, and Clinical Events. J Am Coll Cardiol (2020) 58:2047–67. 10.1016/j.jacc.2011.06.063 22051327

[78] Sabhatini M Apicella L Cataldi M Maresca I Nastasi A Vitale S Effects of a Diet Rich in N-3 Polyunsaturated Fatty Acids on Systemic Inflammation in Renal Transplant Recipients. J Am Coll Nutr (2013) 32(6):376–83. 10.1080/07315724.2013.826482 24606710

[79] Kidney Disease Improving Global Outcomes (KDIGO). Clinical Practice Guideline for the Evaluation and Management of Chronic Kidney Disease. Kidney Int Suppl (2012) 3(1):S1–S150. 10.7326/0003-4819-158-11-201306040-00007

[80] Afsar B Afsar RE Caliskan Y Lentine KL. A Holistic Review of Sodium Intake in Kidney Transplant Patients: More Questions Than Answers. Transplant Rev (2024) 38:100859. 10.1016/j.trre.2024.100859 38749098

[81] Ramesh Prasad GV Nash MM Huang M Zaltzman JS. The Role of Dietary Cations in the Blood Pressure of Renal Transplant Recipients. Clin Transpl (2006) 20:37–42. 10.1111/j.1399-0012.2005.00437.x 16556151

[82] Prasad GVHM Nash MM Zaltzman JS. Role of Dietary Salt Intake in Posttransplant Hypertension with Tacrolimus-Based Immunosuppression. Transpl Proc (2005) 37:1896–7. 10.1016/j.transproceed.2005.04.002 15919496

[83] Moeller T Buhl M Schorr U Distler A Sharma AM. Salt Intake and Hypertension in Renal Transplant Patients. Clin Nephrol (2000) 53(3):159–63. 10749292

[84] Sanders PW Gibbs CL Akhi KM MacMillan-Crow LA Zinn KR Chen YF Increased Dietary Salt Accelerates Chronic Allograft Nephropathy in Rats. Kidney Int (2001) 59:1149–57. 10.1046/j.1523-1755.2001.0590031149.x 11231373

[85] De Keijzer MH Provoost AP Wolff ED Kort WJ Weijma IM Van Aken M The Effect of a Reduced Sodium Intake on Post-renal Transplantation Hypertension in Rats. Clin Sci (1984) 66:269–76. 10.1042/cs0660269 6362959

[86] Soypacaci ZSS Yıldız EA Yıldız EA Keven K Kutlay S Erturk S Effect of Daily Sodium Intake on Post-transplant Hypertension in Kidney Allograft Recipients. Transpl Proc (2013) 45:940–3. 10.1016/j.transproceed.2013.02.050 23622593

[87] Saint-Remy ASM Gellner K Weekers L Bonvoisin C Krzesinski JM. Urinary and Dietary Sodium and Potassium Associated With Blood Pressure Control in Treated Hypertensive Kidney Transplant Recipients: An Observational Study. BMC Nephrol (2021) 13:121. 10.1186/1471-2369-13-121 PMC3506486 23013269

[88] Rodrigo E Monfá E Albines Z Serrano M Fernandez-Fresnedo G Ruiz JC Sodium Excretion Pattern at 1 Year after Kidney Transplantation and High Blood Pressure. Ann Transpl (2015) 20:569–75. 10.12659/AOT.893862 26400681

[89] Hill MA Zhang L Yang Y Sun Z Jia G Parrish AR Insulin Resistance, Cardiovascular Stiffening and Cardiovascular Disease. Metabolism (2021) 2021(119)–154766. 10.1016/j.metabol.2021.154766 33766485

[90] Sechi LAMA Tedde R. Insulin Hypersecretion: A Potential Role in Essential but Not Secondary Hypertension. Metabolism (1992) 41:1261–6. 10.1016/0026-0495(92)90019-7 1435300

[91] De Fronzo RA. The Effect of Insulin on Renal Sodium Metabolism. A Review With Clinical Implications. Diabetologia (1981) 21:165–71. 10.1007/BF00252649 7028550

[92] de Albuquerque Rocha N Neeland IJ McCullough PA Toto RD McGuire DK. Effects of Sodium Glucose Co-transporter 2 Inhibitors on the Kidney. Diab Vasc Dis Res (2018) 15:375–86. 10.1177/1479164118783756 29963920

[93] Ponticelli C Cucchiar D Graziani G. Hypertension after Kidney Transplantation. Transpl Int (2011) 24(5):523–33. 10.1111/j.1432-2277.2011.01242.x 21382101

[94] Almalki B Cunningham K Kapugi M Kane C Agrawal A. Management of Hyperkalemia: A Focus on Kidney Transplant Recipients. Transplatation Rev (2020) 35:100611. 10.1016/j.trre.2021.100611 33711778

[95] Jones J Gruessner RW Gores PF Matas AJ. Hypoaldosteronemic Hyporeninemic Hyperkalemia after Renal Transplantation. Transplantation (1993) 56:1013–5. 8212180

[96] Almolki B Cunningham K Kapugi M Kane C Agrawal A. Management of Hyperkalemia: A Focus on Kidney Transplant Recipients. Transplant Rev (2021) 35(2):100611. 10.1016/j.trre.2021.100611 33711778

[97] Aboghanem A Ramesh Prasad GV. Disorders of Potassium Homeostasis after Kidney Transplantation. World J Transpl (2024) 14(3):95905. 10.5500/wjt.v14.i3.95905 PMC11317851 39295980

[98] Suenaga T Tanaka S Kitamura H Parthasarathy R Tsuruya K Nakano T Kitazono T. Estimated Potassium Intake and the Progression of Chronic Kidney Disease. Nephrol Dialysis Transplantation (2024). gfae277. 10.1093/ndt/gfae277 PMC12207611 39603832

[99] Pochineni VR-BH. Electrolyte and Acid-Base Disorders in the Renal Transplant Recipient. Front Med (2018) 5:261. 10.3389/fmed.2018.00261 PMC6176109 30333977

[100] Palmer B Clegg DJ. Electrolyte and Acid-Base Disturbances in Patients with Diabetes Mellitus. New Engl J Med (2017) 376(2):183–92. 10.1056/NEJMra1503102 26244308

[101] Institute of Medicine. (IoM). Dietary Reference Intakes for Energy, Carbohydrate, Fiber, Fat, Fatty Acids, Cholesterol, Protein, and Amino Acids. Washington, DC: National Academies Press (2005).

[102] World Health Organisation (WHO). Guidelines: Potassium Intake for Adults and Children. (2012). 23617019

[103] K/DOQI. National Kidney Foundation Clinical Practice Guidelines for Nutrition in Chronic Renal Failure. Am J Kidney Dis (2000) 35:S1–S140. 10.1053/j.ajkd.2010.03.022 10895784

[104] Kalantar-Zadeh KFD. Nutritional Management of Chronic Kidney Disease. New Engl J Med (2017) 377:1765–76. 10.1056/NEJMra1700312 29091561

[105] De Nicola L Garofalo C Borrelli S Minutolo R. Recommendations on Nutritional Intake of Potassium in CKD It’s Time to Be More Flexible!. Kidney Int (2022) 102:4:700–73. 10.1016/j.kint.2022.04.046 36150763

[106] Ramos CI González-Ortiz A Espinosa-Cuevas A Avesani CM Carrero JJ Cuppari L. Does Dietary Potassium Intake Associate with Hyperkalemia in Patients with Chronic Kidney Disease? Nephrol Dial Transplant (2021) 11:2049–57. 10.1093/ndt/gfaa232 33247727

[107] Noori N Kalantar-Zadeh K Kovesdy CP Murali SB Bross R Nissenson AR Dietary Potassium Intake and Mortality in Long-Term Hemodialysis Patients. Am J Kidney Dis (2010) 56:338–47. 10.1053/j.ajkd.2010.03.022 20580474 PMC2910783

[108] Cupisti A Kovesdy CP D’Alessandro C Kalantar-Zadeh K. Dietary Approach to Recurrent or Chronic Hyperkalaemia in Patients with Decreased Kidney Function. Nutrients (2018) 2018(10):261. 10.3390/nu10030261 PMC5872679 29495340

[109] Chen YY Chang X Ye Q Liu JR Vaziri ND Guo Y Microbiome-metabolome Reveals the Contribution of Gut-Kidney axis on Kidney Disease. J Transl Med (2019) 17:5. 10.1186/s12967-018-1756-4 30602367 PMC6317198

[110] Gungor O Kircelli F Turan MN Cetin O Elbi H Tatar E Irritable Bowel Syndrome in Renal Transplant Patients: Prevalence, Link with Quality of Life, Anxiety and Depression. Ren Fail (2012) 34:876–9. 10.3109/0886022X.2012.690805 22680982

[111] Ikizler TA Burrowes JD Byham-Gray LD Campbell KL Carrero JJ Chan W KDOQI Clinical Practice Guideline for Nutrition in CKD: 2020 Update. Am J Kidney Dis (2020) 76(Suppl. 1):S1-S107–S107. 10.1053/j.ajkd.2020.05.006 32829751

[112] Nishide Kg Coreoliango-Ring L Rangel EB Rangel ÉB. Hyperkalemia in Diabetes Mellitus Setting. Diseases (2022) 10(2):20. 10.3390/diseases10020020 35466190 PMC9036284

[113] Paoli ABA Bianco A Moro T Mota JF Coelho-Ravagnani CF. The Effect of Ketogenic Diet on Insulin Sensitivity and Weight Loss: Which Came First: The Chicken or Egg? Nutrients (2023) 15(14):3120. 10.3390/nu15143120 37513538 PMC10385501

[114] Gomes V Ferreira F Guerra J Bugalho MJ. New-onset Diabetes after Kidney Transplantation: Incidence and Associated Factors. Word J Diabetes (2018) 9:132–7. 10.4239/wjd.v9.i7.132 PMC6068739 30079149

[115] Kidney Disease Improving Global Outcomes (KDIGO). Clinical Practice Guideline for Diabetes Management in Chronic Kidney Disease. KDIGO, Public Rev. (2023) 98(4) S1–S115. 10.1016/j.kint.2020.06.019 32998798

[116] Querings K Girndt M Geisel J Georg T Tilgen W Reichrath J. 25-hydroxyvitamin D Deficiency in Renal Transplant Recipients. J Clin Endocrinol Metab (2016) 91:526–9. 10.1210/jc.2005-0547 16303843

[117] Filipov J Zlatkov BK Dimitrov EP Svinarov D. Relationship between Vitamin D Status and Immunosuppressive Therapy in Kidney Transplant Recipients. Biotechnol and Biotech Equip (2015) 29:331–5. 10.1080/13102818.2014.995415 PMC4433925 26019648

[118] Cianciolo G Galassi A Capelli I Angelini ML La Manna G Cozzolino M. Vitamin D in Kidney Transplant Recipients: Mechanisms and Therapy. Am J Nephrol (2016) 43(6):397–407. 10.1159/000446863 27229347

[119] Bai YJ Li YM Hu SM Zou YG An YF Wang LL Vitamin D Supplementation Reduced Blood Inflammatory Cytokines Expression and Improved Graft Function in Kidney Transplant Recipients. Immunol Transplant (2023). 14:1152295. 10.3389/fimmu.2023.1152295 PMC10358325 37483634

[120] Koimtzis G Stefanopoulos L Brooker V Geropoulos G Chalklin CG Gupta S The Role of Vitamin D in Kidney Transplantation Outcomes: A Systematic Review. Life Basel (2022) 20(10):1664. 10.3390/life12101664 PMC9605053 36295099

[121] Ewers B Gosbjerg A Moelgaard C Frederiksen AM Marckmann P. Vitamin D Status in Kidney Transplant Patients: Need for Intensified Routine Supplementation. Am J Clin Nutr (2008) 87(2):432–7. 10.1093/ajcn/87.2.431 18258635

[122] Christakos P Dhawan A Overstuff L Verlinden L Carmeliet G. Vitamin D: Metabolism, Molecular Mechanism of Action, and Pleiotropic Effects. Physiol Rev (2016) 96(2016):365–408. 10.1152/physrev.00014.2015 26681795 PMC4839493

[123] Ponticelli C Favi E Ferranresso M. New Onset Diabetes after Kidney Transplantation. Medicina Kaunas (2021) 57(3):250. 10.3390/medicina57030250 33800138 PMC7998982

[124] Wang M Chen Z Hu Y Wang Y Wu Y Lian F The Effects of Vitamin D Supplementation on Glycemic Control and Maternal-Neonatal Outcomes in Women with Established Gestational Diabetes Mellitus: A Systematic Review and Meta-Analysis. Clin Nutr (2021) 40:3148–57. 10.1016/j.clnu.2020.12.016 33386179

[125] Keyzer CA Riphagen IJ Joosten MM Navis G Muller Kobold AC Kema IP Associations of 25(OH) and 1,25(OH)2 Vitamin D with Long-Term Outcomes in Stable Renal Transplant Recipients. J Clin Endocrinol Metab (2015) 100:81–9. 10.1210/jc.2014-3012 25361179

[126] Rojas-Rivera JDLPC Ramos A Ortiz A Egido J. The Expanding Spectrum of Biological Actions of Vitamin D. Nephrol Dial Transpl (2010) 25:2850–65. 10.1093/ndt/gfq313 20525641

[127] Stavroulopoulos A Cassidy MJ Porter CJ Hosking DJ Roe SD. Vitamin D Status in Renal Transplant Recipients. Am J Transpl (2007) 7:2546–52. 10.1111/j.1600-6143.2007.01978.x 17908281

[128] Courbebaisse M Bourmaud A Souberbielle JC Sberro-Soussan R Moal V Le Meur Y Non-skeletal and Skeletal Effects of High Doses versus Low Doses of Vitamin D3 in Renal Transplant Recipients: Results of the VITALE (VITamin D Supplementation in renAL Transplant Recipients) Study, a Randomized Clinical Trial. Am J Transplant (2021) 23:366–76. 10.1016/j.ajt.2022.12.007 36695682

[129] Fonseca I Reguengo H Almeida M Geropoulos G Chalklin CG Gupta S Oxidative Stress in Kidney Transplantation. Transplantation (2014) 97:1058–65. 10.3390/life12101664 24406454

[130] Heldal TF Åsberg A Ueland T Reisaeter AV Pischke SE Mollnes TE Inflammation in the Early Phase after Kidney Transplantation Is Associated with Increased Long-Term All-Cause Mortality. Am J Transpl (2022) 22:2016–27. 10.1111/ajt.17047 PMC9540645 35352462

[131] Dave KN Patil RS. Biological Importance of Ascorbic Acid (Vitamin C) in Human Health-A Classic Review. Int J Res Pharm (2017) 31(7):1e8. 10.1111/ajt.17047

[132] Rho MR Lim JH Park JH Han SS Kim YS Lee YH Evaluation of Nutrient Intake in Early Post Kidney Transplant Recipients. Clin Nutr Res (2013) 2(1):1–11. 10.7762/cnr.2013.2.1.1 23429928 PMC3572820

[133] Huijskens MJ Walczak M Koller N Briedé JJ Senden-Gijsbers BLMG Schnijderberg MC Technical Advance: Ascorbic Acid Induces Development of Double-Positive T Cells from Human Hematopoietic Stem Cells in the Absence of Stromal Cells. J Leukoc Biol (2014) 96:1165–75. 10.1189/jlb.1TA0214-121RR 25157026

[134] Esteban MA Wang T Qin B Yang J Qin D Cai J Vitamin C Enhances the Generation of Mouse and Human Induced Pluripotent Stem Cells. Cell Stem Cell (2010) 6:71–9. 10.1016/j.stem.2009.12.001 20036631

[135] Yepes-Calderon M Van den Veen Y del CFM Kremer D Sotomayor CG Knobbe TJ Vitamin C Deficiency After Kidney Transplantation: A Cohort and Cross-Sectional Study of the Transplant Lines Biobank. Eur J Nur (2024) 63(6):2357–66. 10.1007/s00394-024-03426-7 PMC11377669 38811416

[136] Borran M Dashti-Khavidaki S Alamdari A Naderi N. Vitamin C and Kidney Transplantation: Nutritional Status, Potential Efficacy, Safety, and Interactions. Clin Nutr ASPEN (2021) 41:1–9. 10.1016/j.clnesp.2020.12.017 33487249

[137] Ferraro PM Curhan GC Gambaro G Taylor EN. Total, Dietary and Supplemental Vitamin C Intake and Risk of Incident Kidney Stones. Am J Kidney Dis (2016) 67(3):400–7. 10.1053/j.ajkd.2015.09.005 26463139 PMC4769668

[138] Thomas L Elinder CG Tiselius HG Wolk A Akesson A. Ascorbic Acid Supplements and Kidney Stone Incidence Among Men: A Prospective Study. JAMA Intern Med (2016) 173:386–8. 10.1001/jamainternmed.2013.2296 23381591

[139] Khoshnam-Rad N Khalili H. Safety of Vitamin C in Sepsis: A Neglected Topic. Curr Opin Crit Care (2019) 25:329–33. 10.1097/MCC.0000000000000622 31107310

[140] Kabelitz D Cierna L Juraske C Zarobkiewicz M Schamel WW Peters C. Empowering T Cell Functionality with Vitamin C. Eur J Immunol (2024) 54:e2451028. 10.1002/eji.202451028 38616772

[141] Wilson JX. Mechanism of Action of Vitamin C in Sepsis: Ascorbate Modulates Redox Signaling in Endothelium. Biofactors (2009) 2009(35):5–13. 10.1002/biof.7 PMC2767105 19319840

[142] Boran M Dashti-Khavdaki S Alamdari A Naderi N Minoo F. Evaluation of the Effect of High Dose Intravenous Vitamin C on Delayed Allograft Function in Deceased Donor Kidney Transplantation: A Preliminary Report. Ren Replace Ther (2020) 6(31):31. 10.1186/s41100-020-00279-8

[143] Hahn M Wood A Hasse JM. Nutrition Support Management of Organ Transplant Recipients in the Acute Posttransplant Phase. Nutr Clin Pract (2023) 39:45–58. 10.1002/ncp.11104 38081296

[144] Hendricks F Smeets JS Broers JH van Kranenburg JMX van der Sande FM Kooman JP End Stage Renal Disease Patients Lose a Substantial Amount of Amino Acids during Hemodialysis. J Nutrit (2020) 1160–6. 10.1093/jn/nxaa010 32006029 PMC7198312

[145] Carminatti M Tedesco-Silva H Fernandes NMS Sanders-Pinheiro H. Chronic Kidney Disease Progression in Kidney Transplant Recipients: A Focus on Traditional Risk Factors. Nephrology (2019) 24(2):141–7. 10.1111/nep.13483 30159972

[146] Lopes IM Martín M Ferrasti P Martínez JA. Benefits of a Dietary Intervention on Weight Loss, Body Composition and Lipid Profile after Renal Transplantation. Nutrition (1999) 15:1. 10.1016/s0899-9007(98)00137-3 9918055

[147] Kramer H. Diet and Chronic Kidney Disease. Adv Nutr (2019) 2019(Suppl. l):4S367–79. 10.1093/advances/nmz011 PMC6855949 31728497

[148] Gelber RP Kurth T Kausz AT Manson JE Buring JE Levey AS Association between Body Mass Index and CKD in Apparently Healthy Men. Am J Kidney Dis (2005) 46:871–80. 10.1053/j.ajkd.2005.08.015 16253727

[149] Anderson CAM Nguyer HA. Nutrition Education in the Care of Patients with Chronic Idney Disease and End Stage Renal Disease. Semin Dial (2018) 2018:1–7. 10.1111/sdi.12681 29455475

[150] Adams KMKM Powell M Zeisel SH. Nutrition in Medicine: Nutrition Education for Medical Students and Residents. Nutr Clin Pract (2010) 25:471–80. 10.1177/0884533610379606 20962306 PMC4594871

[151] Kushner RF. Barriers to Providing Nutrition Counselling by Physicians: A Survey of Primary Care Practitioners. Prev Med (1995) 24:546–52. 10.1006/pmed.1995.1087 8610076

[152] British Transplant Society (BTS, UK) Guideline for the Management of the Patient with a Failing Kidney Transplant. (2023).

[153] Djamali A Samaniego M Muth B Muehrer R Hofmann RM Pirsch J Medical Care of Kidney Transplant Recipients After the First Posttransplant Year. Clin J Am Soc Nephrol (2006) 1:623–40. 10.2215/CJN.01371005 17699268

